# Diagnostic yield of dental radiography and digital tomosynthesis for the identification of anatomic structures in dogs

**DOI:** 10.3389/fvets.2024.1489239

**Published:** 2024-11-20

**Authors:** Tanner May, Milinda Jean Lommer, Boaz Arzi, Stephanie Lynne Goldschmidt, David C. Hatcher, Maria M. Soltero-Rivera

**Affiliations:** ^1^Aggie Animal Dental Center, Mill Valley, CA, United States; ^2^School of Veterinary Medicine, Veterinary Surgical and Radiological Sciences, University of California, Davis, Davis, CA, United States

**Keywords:** oral anatomy, dogs, digital tomosynthesis, dentition, dental radiography, imaging

## Abstract

**Introduction:**

The purpose of this study was to evaluate the use of a novel imaging modality, digital tomosynthesis (DT), for identification of predefined anatomic dental and maxillomandibular structures in dogs.

**Methods:**

DT images were compared to conventional intraoral dental radiography (DR) for the diagnostic yield regarding the presence and quality of visualization of 35 structures. DT imaging and full mouth DR were obtained on 16 canine cadaver heads and a semi-quantitative scoring system was used to characterize the ability of each imaging method to identify the anatomic structures.

**Results:**

The results demonstrated that each imaging modality, and orientation, was superior for certain anatomic structures.

**Discussion:**

Overall, although one modality did not prove superior to the other, digital tomosynthesis appears to be an appropriate novel tool for identification of specific anatomic structures in the dog skull.

## Introduction

Intraoral dental radiography (DR) has long been considered the gold standard imaging modality to visualize dental and osseous structures beneath the gingival margin during an anesthetized procedure. Previous studies have determined the diagnostic yield of canine DR to be high, justifying the routine use in dogs (1). A standard full-mouth set of DR images is 10–14 views, showing all the teeth with 2–3 mm of the periapical area visible for each root (2). Yet, DR is limited by the fact that accurate representation of the teeth requires technical skill in acquiring each image and a 2-D image may contain structure superimposition that makes evaluation of certain landmarks or alveolar structures difficult (3). Further, while tube shifting allows for tangential assessment of differing areas of the structure, the 2-D image of a 3-D object allows only, almost exclusively, evaluation of the mesial and distal aspects of a tooth (4). Finally, acquiring full mouth DR studies can take a variable amount of time, all occurring while the patient is under general anesthesia. In a veterinary hospital setting, acquiring a set of full mouth radiographs is highly dependent upon many factors including the experience of the person obtaining the series, the size and shape of the patient's head, and the size of the x-ray sensors or phospor plates being used. While the length of time to interpret DR images compared to DT images may be similar; the additional time under anesthesia to acquire the images, while justified to gain the diagnostic knowledge, is not without risk, particularly if the patient is not tolerating the anesthesia well (5). Radiation levels are also an important factor to consider as intraoral DR levels have been shown to range from 1 to 20 μSv (6); while the effective doses for cone-beam computed tomography (CBCT) and DT studies were 30 and 65 μSv, respectively (7).

**Figure 1:**
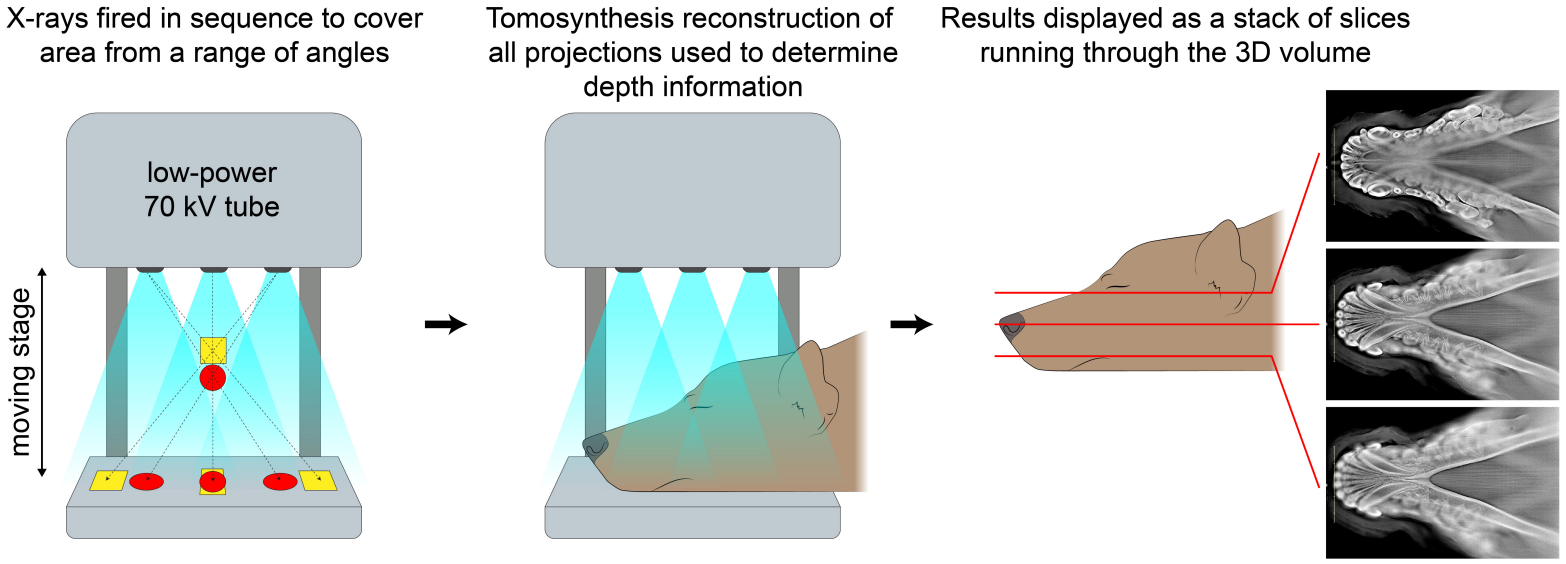
Schematic showing how images are obtained, reconstructed, and displayed in digital tomosynthesis.

Digital tomosynthesis (DT) is a relatively newer imaging modality that has not been fully validated for use in veterinary dentistry. DT is able to create a pseudo-3-D image series by obtaining multiple 2-D radiographs at different angles to generate cross-sectional images that are compiled together for interpretation (Figure 1). In previous human studies, DT has shown diagnostic value in imaging of the breast (8, 9), chest (10), head and neck (11), and the musculoskeletal system (12–15). A recent study evaluating the use of DT for dental radiography in cats demonstrated a significantly higher diagnostic yield for relevant anatomic landmarks, when compared to DR, with the primary advantage of DT being its ability to eliminate superimposition (16). The objective of this study was to evaluate the diagnostic yield of DT imaging compared to standardized intraoral DR for evaluating predefined anatomic landmarks of both teeth and bone in dogs.

The feline study evaluated two orientations of head placement, lateral and dorsoventral, for their combined score compared to DR. With the goal to reduce overall radiation, time to acquire the complete diagnostic set of images, and the total time of anesthesia, this study of the canine head evaluated both orientations separately to see if one would prove superior to the other. The study in the cat cadaver heads also evaluated each tooth as a whole, rather than each root structure as its own entity. Individual roots of multi-rooted teeth were assessed separately in this canine study due to the influence that individual root pathology can have on treatment planning. Thirty-five anatomic structures were evaluated using DT, in lateral and dorsoventral orientation, and DR. These landmarks were selected based on limitations previously identified in DR, but also for areas that are routinely evaluated due to common dental diseases. We hypothesized that DT, in either orientation, would not be inferior to DR in locating and visualizing clinically relevant anatomic structures on the skull of dogs.

## Materials and methods

### Animals

Sixteen canine cadaver, non-brachycephalic heads of unknown breed and sex were evaluated. The dogs were euthanized for reasons unrelated to this study. Once obtained, the heads were screened by a resident in small animal veterinary dentistry (TM) for grossly obvious periodontal or endodontic diseases as well as congenital or acquired maxillofacial pathology that would impact anatomic quality. All specimens were determined to be free of observable pathosis.

### Image acquisition

Full-mouth intraoral digital radiographic (DR) studies (10 films per head) were obtained using an indirect digital imaging system (Heliodent MD, Siemens Sirona; ScanX, Air Techniques) at 60 kVp, 7 mA, and exposure times of 0.2–0.4 s (depending on the location of evaluated teeth). The system yielded a resolution of up to 18 linepairs/mm, which equated to a pixel size of 55.5 μm. Radiographic images included the standard series of views in accordance with American Veterinary Dental College^®^ guidelines. Table 1 displays which views were utilized to evaluate each of the 35 anatomic landmarks (Table 1).

**Table 1:** Anatomical landmarks evaluated in the study and corresponding dental radiographic (DR) views used to assess each of these, bilaterally.

**Anatomic landmark**	**DR view(s)**	**Anatomic landmark**	**DR view(s)**
Mandibular canine dentoalveolar structures	Mandibular incisor teeth; occlusal	Nasolacrimal canal	Maxillary premolar/molar teeth; lateral
	Mandibular canine teeth; lateral		
Mandibular first molar dentoalveolar structures	Mandibular premolar/molar teeth; lateral	Plane of the hard Palate	Maxillary canine teeth; lateral
			Maxillary premolar/molar teeth; lateral
Maxillary canine dentoalveolar structures	Maxillary incisor teeth; occlusal	Major Palatine Foramen	Maxillary premolar/molar teeth; lateral
	Maxillary canine teeth; occlusal		
Maxillary fourth premolar dentoalveolar structures	Maxillary premolar/molar teeth; lateral	Palatine Fissures	Maxillary incisor teeth; occlusal
Maxillary first molar dentoalveolar structures	Maxillary premolar/molar teeth; lateral	Nasal Turbinates	Maxillary incisor teeth; occlusal
Middle mental foramen	Mandibular incisor teeth; occlusal	Mandibular Symphysis	Mandibular incisor teeth; occlusal
	Mandibular canine teeth; lateral		
Caudal mental foramen	Mandibular canine teeth; lateral	Ventral Rim of the Orbit	Maxillary premolar/molar teeth; lateral
Infraorbital foramen	Maxillary canine teeth; lateral	Zygomatic Arch	Maxillary premolar/molar teeth; lateral
	Maxillary premolar/molar teeth; lateral	Incisivomaxillary canal	Maxillary canine teeth; lateral

Digital tomosynthesis (DT) imaging was obtained using an Adaptix 3D X-ray Small Animal Imaging system. A dorsoventral (DV) study and a right lateral (right side down) study were obtained for each head. Serial slices of the heads were obtained such that the height of the skull (rounded up to the nearest 10 mm) was divided into 50 evenly spaced slices to create the study. The DT system has a pixel size of 99 μm, which yields a resolution of up to 5 linepairs/mm. For the DV studies, the cadaver heads were placed with the plane of the palate parallel to the flat panel beneath the cadaver and the crosshairs aligned sagittal over the midline. For the right lateral studies, a wedge was placed beneath the muzzle to hold the sagittal plane of the head parallel to the flat panel beneath and the crosshairs aligned to the plane of the palate. The DR and DT images were obtained by a single investigator (TM), with optimization and input from a board-certified veterinary dentist (MSR).

### Image evaluation and scoring

**Figure 2:**
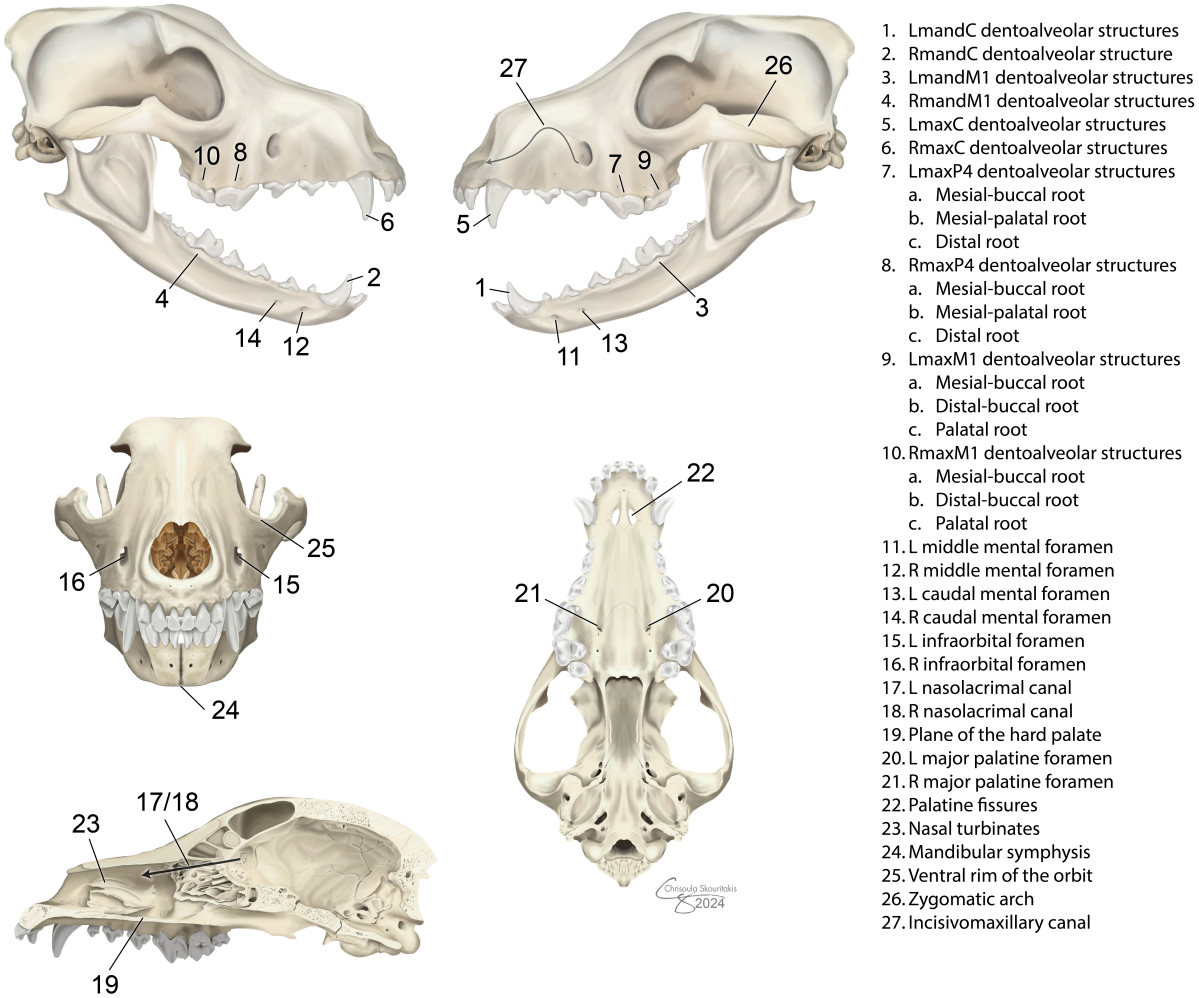
Predefined anatomic structures evaluated in dogs using dental radiography and digital tomosynthesis.

DR, dorsoventral DT, and lateral DT were evaluated separately for identification and quality of the 35 predefined anatomic structures (Figure 2). The DR images were uploaded to a confidential repository for remote evaluation. The DT images were stored on an online image viewer hosted by Adaptix. DR images were randomized and both imaging modalities were evaluated by 2 board-certified veterinary dentists (ML, MSR) and a resident in small animal dentistry (TM). Evaluators were blinded and worked independently. DT images were evaluated before DR to avoid bias.

A semi-quantitative scoring system was used for each imaging method, including the separate judging of the DV and lateral DT studies. Scoring was on a scale of 0–1 for the presence of the structure (0 = unable to identify, 1 = able to identify), and on a scale of 0–3 for quality of identification (0 = unable to identify, 1 = poor visualization, 2 = fair visualization, 3 = excellent visualization). Mean scores over the 16 cadaver heads were calculated for each anomic structure using each imaging method; separate for the DV and lateral DT studies. A total mean score of the imaging method for evaluating all 35 anatomic structures was calculated (17).

### Statistical analysis

The results and mean scores were reported as mean ± SE. For each region, scores evaluated by each grader, and each imaging modality were used to calculate the overall mean ± SE by taking the average score from all three raters. A Friedman test (for the omnibus test) was used to evaluate differences between the two modalities and the two orientations of DT. For *post-hoc* comparisons, a Nemenyi's procedure all pairwise comparison of modalities was then used to determine statistical significance. Significance was set at values of *p* < 0.05. Kappa agreement coefficient for inter-rater agreement could not be assessed due to sample size and occurrence of perfect agreement between raters. Instead, the percentage of occurrence of the same score for raters per landmark and specimen was calculated for each imaging method.

### Maxillary width

Each of the cadaver heads was measured for the distance between the pulp cavities of the mesial-buccal roots of the maxillary first molar teeth. The widths ranged from 5.55 to 9.33 cm, with a mean of 7.62 cm and median of 7.83 cm. This measurement was input as the “maxillary width” and was used to determine whether the quality scores varied in relation to the size of the head. The average scores were then plotted, and a non-parametric slope estimate (Theil-Sen) was calculated for each anatomic structure for each modality. *P*-values of the non-parametric slope estimate were calculated under the null hypothesis of no association i.e., a slope of zero.

## Results

### Animals

No information regarding sex, breed, or age was provided for the 16 canine cadaver heads used. However, all heads had characteristic appearance of medium- to large-sized dogs with mesocephalic skull conformation. Based on the pulp to tooth width ratio measured on obtained images, the 16 cadaver heads were approximated to be consistent with one juvenile (< 12 months), nine young adult (12–32 months), and six mature adults (>32 months) (18).

### Overall scores

**Figure 3:**
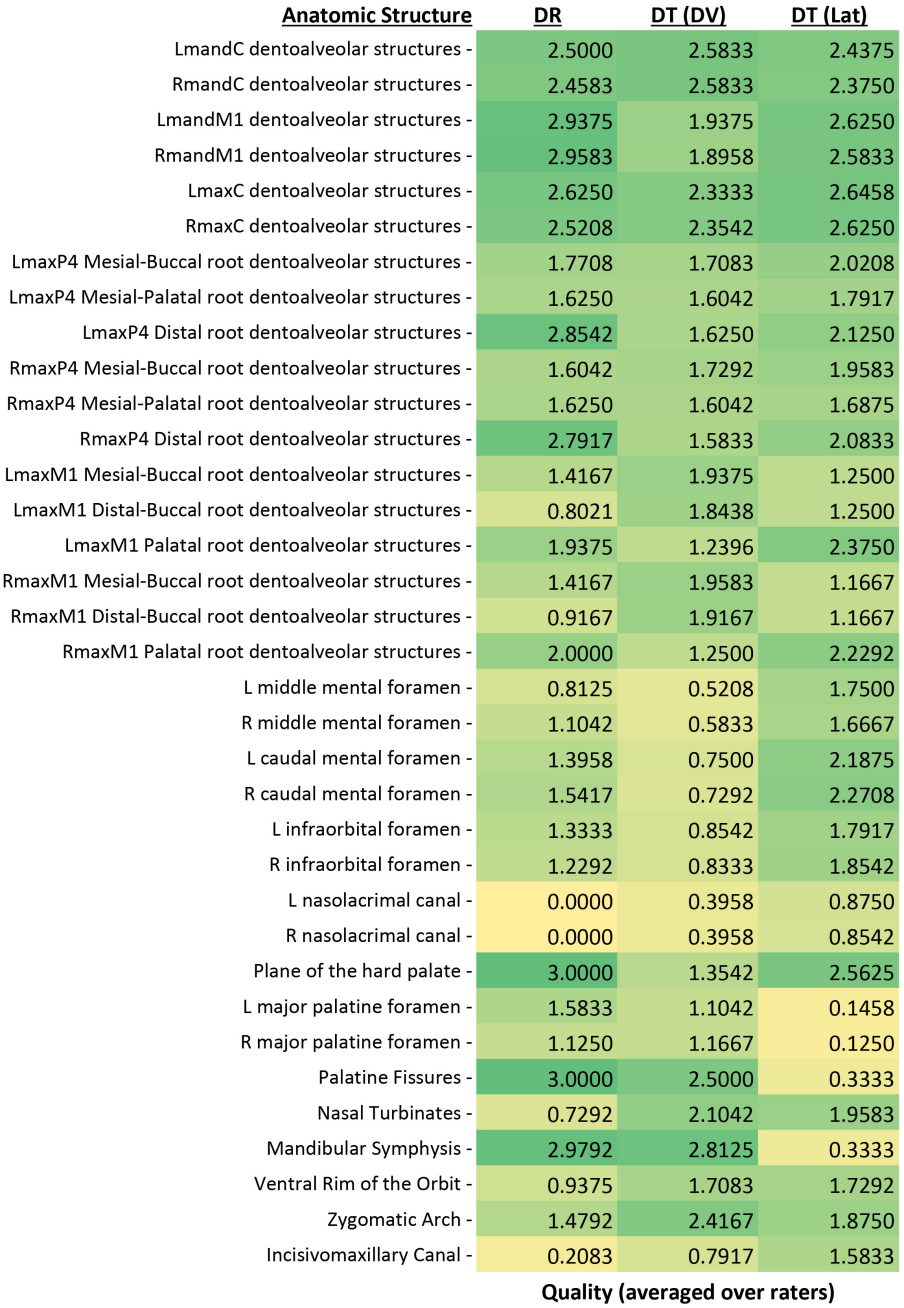
Mean combined scores for all graders for each of the 35 anatomic structures evaluated with both methods and orientations. Scores were assigned by use of a scale of 0–3 as follows: 0 = inability to identify the anatomic structure, 1 = poor identification of anatomic structure, 2 = good identification of anatomic structure, and 3 = excellent identification of anatomic structure. This heat map displays green as the highest quality value (3) and yellow as the lowest quality value (0).

**Figure 4:**
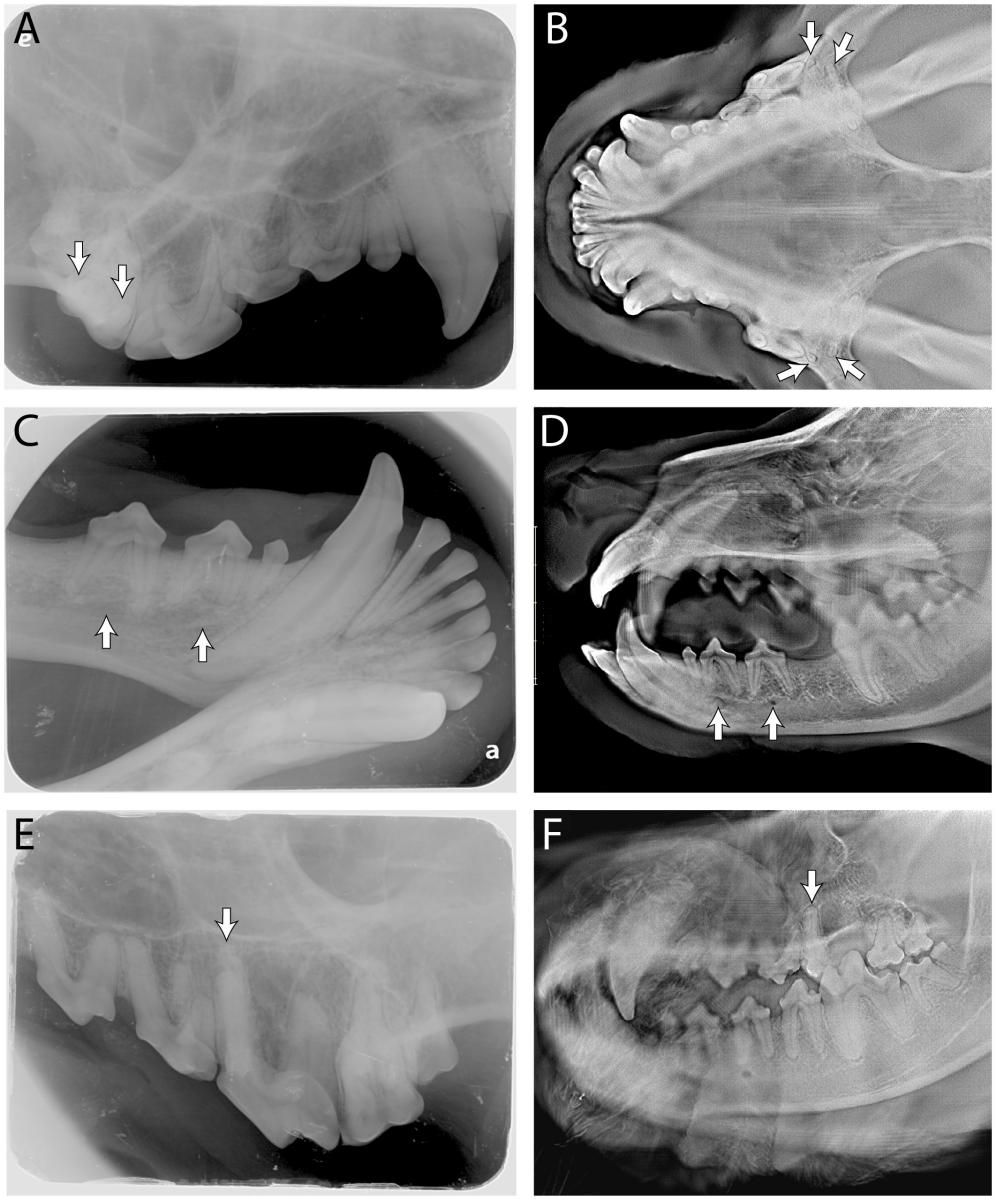
Side by side comparison of the buccal roots of the maxillary first molar indicated by the white arrows in **(A**, DR**)** and **(B**, DT-DV**)**; side by side comparison of the middle and caudal mental foramen indicated by the white arrows in **(C**, DR**)** and **(D**, DT-lateral**)**; side by side comparison of the mesial-palatal root of the right maxillary fourth premolar indicated by the white arrow in **(E**, DR**)** and **(F**, DT-lateral**)**. The “e” and “a” on **(A, C)**, respectively, are identification marks on the phosphor plates to help with orientation and can be ignored.

Figure 3 displays average ratings for each of the 35 anatomic structures by each individual imaging modality (and orientation; Figure 3). Each imaging modality—digital radiographs (DR), lateral digital tomosynthesis (DT), and dorsoventral DT—showed superior performance in capturing different structures, highlighting their unique strengths without one clearly overshadowing the others. Structures that scored statistically similarly between the two modalities and the two orientations of DT included the root of the left mandibular canine tooth, the mesial-buccal root of the left maxillary fourth premolar tooth, and bilateral mesial-palatal roots of the maxillary fourth premolar teeth. Structures that were scored with the highest quality on DR included bilateral roots of the mandibular first molar teeth, bilateral distal roots of the maxillary fourth premolar teeth, the plane of the hard palate and the palatine fissures. Structures that scored the highest on lateral DT included bilateral middle mental foramina, bilateral caudal mental foramina, and the incisivomaxillary canal. Structures that had the highest quality scores for dorsoventral DT included bilateral mesial-buccal roots of the maxillary first molar teeth, bilateral distal-buccal roots of the maxillary first molar teeth, and the zygomatic arch. Figure 4 shows examples of anatomic landmarks that had higher quality scores on DT when compared to DR (Figure 4).

Structures that had the highest quality scores with DR and lateral DT included bilateral roots of the maxillary canine teeth, bilateral palatal roots of the maxillary first molar teeth, bilateral caudal mental foramina, and bilateral infraorbital foramina. Structures that had the highest quality scores with DR and dorsoventral DT included the root of the right mandibular canine tooth, bilateral major palatine foramina, and the mandibular symphysis. Structures that had the highest quality scores with lateral DT and dorsoventral DT included the mesial-buccal root of the right maxillary fourth premolar tooth, the nasal turbinates, and the ventral rim of the orbit (Table 2).

**Table 2:** Anatomical landmarks evaluated in the study and corresponding imaging modality or orientation which had the highest quality grade.

**Structure**	**View(s)**	**Structure**	**View(s)**	**Structure**	**View(s)**
LmandC	All modalities similar	RmandC	Dorsoventral DT and DR	L middle mental	Lateral DT
LmandM1	DR	RmandM1	DR	R middle mental	Lateral DT
LmaxC	Lateral DT and DR	RmaxC	Lateral DT and DR	L caudal mental	Lateral DT and DR
LmaxP4 MB	All modalities similar	RmaxP4 MB	Lateral DT and Dorsoventral DT	R caudal mental	Lateral DT and DR
LmaxP4 MP	All modalities similar	RmaxP4 MP	All modalities similar	L infraorbital	Lateral DT and DR
LmaxP4 D	DR	RmaxP4 D	DR	R infraorbital	Lateral DT and DR
LmaxM1 MB	Dorsoventral DT	RmaxM1 MB	Dorsoventral DT	L nasolacrimal	Lateral DT
LmaxM1 DB	Dorsoventral DT	RmaxM1 DB	Dorsoventral DT	R nasolacrimal	Lateral DT
LmaxM1 P	Lateral DT and DR	RmaxM1 P	Lateral DT and DR	L major palatine	DR and Dorsoventral DT
Nasal Turbinates	Lateral DT and Dorsoventral DT	Zygomatic Arch	Dorsoventral DT	R major palatine	Dorsoventral DT and DR
Mandibular Symphysis	DR and Dorsoventral DT	Ventral Rim of the Orbit	Lateral DT and Dorsoventral DT	Palatine fissures	DR
Incisivomaxillary Canal	Lateral DT			Plane of the Hard Palate	DR

### Influence of maxillary width

Ratings were noted to decrease as the maxillary width increased on lateral DT in structures including the ventral rim of the orbit, right nasolacrimal canal, all three roots of the right maxillary fourth premolar tooth, the mesial-palatal root of the left maxillary fourth premolar tooth, and the incisivomaxillary canal. As maxillary width increased, structures with ratings noted to decrease on the dorsoventral DT included the zygomatic arch and bilateral distal-buccal root of the maxillary first molar tooth. On DR, structures that had a decrease in quality rating with increasing maxillary width included the right major palatine foramen, bilateral mesial-palatal roots of the maxillary fourth premolar teeth, and the left infraorbital canal.

### Rater agreement

As previously stated, kappa agreement was performed but could not be assessed statistically due to the small sample size and instances of perfect rater agreement on certain structures. Two structures in the DR imaging modality, palatine fissures and plane of the hard palate, had complete agreement between the three graders. However, a rater agreement percent was calculated, and the vast majority of agreements were over 80% between the three graders and the total of the averages for each modality can be seen below (Table 3).

**Table 3:** Average rater agreement for a given imaging modality or orientation.

**Imaging modality**	**Overall agreement**
DR	93.0%
DT (Lat)	89.9%
DT (DV)	86.7%

## Discussion

To our knowledge, this is the first study to evaluate the use of digital tomosynthesis (DT) for identification of dental and oromaxillofacial anatomic structures of the dog. A recently published work using the same method, but in the cat, revealed the superior diagnostic yield of DT compared to DR (16). In the dog, however, each imaging modality showed superior performance in capturing different anatomic structures, highlighting their unique strengths. By expanding the anatomic structures evaluated and qualifying the orientations of DT separately, we may be able to gather more specific information about the value and use of DT in veterinary dentistry at a primary and specialty care level.

The lateral orientation of DT was noted to be superior at assessing foramina and canals. The lateral positioning of many of these structures likely enabled their superior visualization when panning through the lateral slices. Foramina and canals are important landmarks to identify when performing local nerve blocks and to avoid when carrying out extractions. The palatal fissures and palatine foramina (which are oriented dorsoventrally opposed to the lateral orientation of other foramina of the skull) scored the lowest on lateral DT as they proved difficult to discern when panning through the images. The DT in the dorsoventral orientation and DR had significantly higher quality scores for the palatal foramina and palatine foramina.

The dorsoventral orientation of DT had higher quality scores for the buccal roots of the maxillary first molar teeth. These results are confounding, as both buccal roots scored highest (1.84–1.93), but the palatal root of this same tooth averaged the lowest scores on dorsoventral DT. The lateral orientation of DT and DR were statistically similar, and superior to dorsoventral DT, when it came to assessing the quality of visualization of this palatal root. Possible causes of this difference may be the length and shape of the buccal roots compared to the palatal root; the longer buccal roots may provide more opportunity for examination compared to the shorter palatal root on the dorsoventral orientation. Also, the wide shape of the palatal root may provide more opportunity for evaluation than the thin buccal roots on the lateral orientation.

Both orientations of DT were able to overcome the summation issue and scored significantly higher in quality compared to DR for the nasal turbinates. Nasal turbinates are known to be quite difficult to assess on intraoral digital radiographs due to summation of structures over the nasal cavity. When looking for origin of rhinitis in dogs on intraoral radiographs, odontogenic sinusitis pathology was frequently missed (19). The diagnostic potential for assessing upper respiratory disease has not, to the authors' knowledge, been tested with digital tomosynthesis, but the ability to pan through the images may allow for a more detailed evaluation of the nasal structures.

Finally, there were structures that had minimal difference between the modalities. The canine teeth all averaged a quality score between 2.3 and 2.6 on the grading scale regardless of which modality was used. It was noted that the maxillary canine teeth were graded the highest on the lateral orientation of DT and the mandibular canine teeth were graded the highest on the dorsoventral orientation of DT. This is likely due to the angle of the root structure and how much of the tooth can be seen on an individual slice while panning through the images. The almost horizontal plane of the mandibular canine teeth root structure allowed dorsoventral DT to view these teeth in almost their entirety in one image while the more vertical positioning of the maxillary canine teeth could be viewed in almost their entirety on the lateral DT. Furthermore, the ability to evaluate the presence of an oronasal fistula at the apex of a canine tooth is limited on digital radiograph (4), but DT could likely overcome the issue created by superimposition.

The mesial-palatal and mesial-buccal roots of the maxillary fourth premolar tooth also scored very similarly between DR and DT. The only noted statistically significant difference was that the right maxillary fourth premolar tooth mesial-buccal roots were scored higher on both the dorsal and lateral DT when compared to DR. It is possible that the head in right lateral recumbency, even with the sagittal plane parallel to the flat panel, allowed for improved visualization of these root structures. As a common tooth to undergo root canal therapy, recognition of the curvature and dilaceration that these roots sometimes display is vital to a successful filing and obturation. In human molar tooth roots, the radiographic length appeared shorter on average than the actual length measured following extraction (20). Sharp curves may increase stress on endodontic files and morphologic variations in the root canals such as long, narrow, or curved canals are more likely to result in endodontic accidents and instrumenting mishaps, such as ledge formation, fractured instruments, blockage of the canal, or zipping (21). Even a slight advantage on imaging of the anatomic shape of these canals, prior to performing endodontic procedures, could help lead to more successful outcomes and less clinician frustration due to said accidents and instrumenting mishaps.

The maxillary width did not seem to substantially affect the quality scoring. Our initial thought was that structures on the periphery of the skull (e.g., the mesial-buccal root of the maxillary fourth premolar tooth and the buccal roots of the maxillary first molar tooth) would score lower as skull size increased due to the limitations of the machine. Overall, we found that the size of the skull only minimally affected the quality scoring. In the time since the data collection stage of this project was performed, the manufacturer of the machine used for DT image acquisition has doubled the size of the detector, halved the acquisition time, and introduced improvements to the acquisition software. It is possible that the latest version of the machine with these updates may eliminate the issues we observed with larger canine heads, especially with the structures that showed decreased quality on the periphery.

Overall agreement for DR was 93.0% while lateral DT was 89.9% and dorsoventral DT was 86.7%. The difference in agreement could be attributed to the raters' overall experience with the given modality. In a recent study designed to assess agreement of interpretation of intraoral DR between veterinary students, veterinary dentistry residents, and veterinary dentistry specialists, it was revealed that agreement was fair to good, while concluding that interpretation of radiographs is highly subjective (22). When asked about the individuals' confidence level, only 20% of early career diplomates stated a high level of mastery, 70% of late career diplomates stated a high level of mastery, and none of the residents stated a high level of mastery (20). Even though intraoral dental radiographs have been used for many years vs. the novel imaging modality of digital tomosynthesis, agreement between different graders may still be variable. It would be expected that a greater amount of experience with digital tomosynthesis over time would lead to an increase in agreement. Future studies involving DT to further assess agreement would likely need to evaluate its use in diagnosing common pathologic changes that are routinely noted on DR, and potentially comparing raters with different levels of experience similar to the study on DR.

The overall time to obtain quality images of an individual canine skull in both orientations of DT was ~5 min. This is substantially faster than obtaining full mouth intraoral digital radiographs on the canine skull. Depending on whether the user has phosphor plates or a direct digital sensor, a complete set of diagnostic quality dental radiographs of the canine skull requires a minimum of 10 radiographs, which can take a variable amount of time depending upon the skill of the person performing the radiographs. All the imaging for the study was acquired by a resident in veterinary dentistry. It could be beneficial in future studies to compare the acquisition and interpretation time with a less experienced operator for both DR and DT to see how this may affect overall time. While this investigation only utilized mesocephalic skulls, acquiring a full-mouth intraoral radiograph survey on patients with different shaped skulls, especially brachycephalic patients, is typically time consuming (and often unrewarding due to superimposition). Further studies on patients with differing skull shape are warranted. Also, implementing the use of DT could substantially reduce the duration of anesthesia for patients in a clinical setting without compromising the quality of diagnostic information obtained. Additionally, studies evaluating the diagnostic yield of DT as compared to DR, with regard to pathologic lesions, are also warranted.

DT provides a consistent image orientation between patients due to the single scan. The ultimate goal of all imaging is to show the anatomic truth of what is not viewable with the physical eye. Repositioning of both the sensor and the cone when acquiring DR images can contribute to each image having different levels of exposure, angulation of the teeth on the film (foreshortening or elongation), and a limited window of viewing. These obliqued images, often used for the caudal maxilla, can change the perceivable length, angle, and shape of teeth and their roots. This information is especially clinically relevant when performing endodontic procedures like root canal therapy as previously discussed. The focal area obtained on DR can also limit the clinician's view of a patient's problems as other teeth, roots, and structures may not be visible on the film acquired for a known problem. Intraoperatively, DR would still have significant benefits in evaluating the progress of procedures including extractions and endodontic therapies, however, the 3D aspect of DT would likely contribute to higher quality postoperative imaging, specifically when evaluating obturation following root canal treatment. DT provides a complete image of oral cavity and surrounding structures that could potentially be vital for early diagnosis of changes related to periodontal disease, endodontic disease, and neoplasia.

## Conclusion

The results of this study have demonstrated that digital tomosynthesis can produce quality images for specific anatomic structures of the dental and oromaxillofacial structures in the dog. None of the imaging modalities (DR vs. DT) or orientations (lateral vs. dorsal DT) was consistently graded higher than the others for all the observed anatomic structures. While this investigation was limited to specimens without evidence of dental diseases, and not all structures were graded higher on DT, the results confirm that DT is a valuable and easy to use tool for visualizing dental and maxillomandibular structures in the dog. Our findings suggest that DT may provide an alternative to DR in veterinary practice, and the efficient acquisition time of digital tomosynthesis has the potential to significantly decrease anesthesia time for patients receiving dental care.

## Data Availability

The original contributions presented in the study are included in the article/supplementary material, further inquiries can be directed to the corresponding author.
